# Peripheral Blood Mononuclear Cell Oxytocin and Vasopressin Receptor Expression Positively Correlates with Social and Behavioral Function in Children with Autism

**DOI:** 10.1038/s41598-019-49617-9

**Published:** 2019-09-17

**Authors:** Irena Voinsky, Sirish C. Bennuri, Julie Svigals, Richard E. Frye, Shannon Rose, David Gurwitz

**Affiliations:** 1 Department of Human Molecular Genetics and Biochemistry, Sackler Faculty of Medicine, Tel Aviv University, Tel Aviv, Israel; 2 Department of Pediatrics, University of Arkansas for Medical Sciences and Arkansas Children’s Research Institute, Little Rock, AR USA; 3 Department of Child Health, University of Arizona College of Medicine-Phoenix, Phoenix, AZ USA; 4 Barrow Neurological Institute at Phoenix Children’s Hospital, Phoenix, AZ USA; 5 Sagol School of Neuroscience, Tel Aviv University, Tel Aviv, Israel

**Keywords:** Autism spectrum disorders, Predictive markers

## Abstract

The peptide hormone oxytocin is an established regulator of social function in mammals, and dysregulated oxytocin signaling is implicated in autism spectrum disorder (ASD). Several clinical trials examining the effects of intranasal oxytocin for improving social and behavioral function in ASD have had mixed or inclusive outcomes. The heterogeneity in clinical trials outcomes may reflect large inter-individual expression variations of the oxytocin and/or vasopressin receptor genes *OXTR* and *AVPR1A*, respectively. To explore this hypothesis we examined the expression of both genes in peripheral blood mononuclear cells (PBMC) from ASD children, their non-ASD siblings, and age-matched neurotypical children aged 3 to 16 years of age as well as datamined published ASD datasets. Both genes were found to have large inter-individual variations. Higher *OXTR* and *AVPR1A* expression was associated with lower Aberrant Behavior Checklist (ABC) scores. *OXTR* expression was associated with less severe behavior and higher adaptive behavior on additional standardized measures. Combining the sum expression levels *OXTR*, *AVPR1A*, and *IGF1* yielded the strongest correlation with ABC scores. We propose that future clinical trials in ASD children with oxytocin, oxytocin mimetics and additional tentative therapeutics should assess the prognostic value of their PBMC mRNA expression of *OXTR*, *AVPR1A*, and *IGF1*.

## Introduction

The peptide hormone oxytocin is strongly implicated in social behavior and is conserved in mammals. Oxytocin has been extensively studied in the context of social function in animals and humans, in particular in children and adults with autism spectrum disorder (ASD)^1–3^. Intranasal oxytocin was shown to improve social function and empathy in healthy individuals^4–6^, and is thus a candidate therapeutic for children with ASD.

It has been suggested that genetic variations of the *OXTR* alleles, promoter methylation, and/or expression levels in brain areas implicated in social function may contribute to the variable response observed in clinical trials with intranasal oxytocin in ASD^7,8^. In addition, differences between and within animal species in sociality may in part be explained by substantial differences in *OXTR* expression levels within the social brain network, such as the amygdala, the anterior cingulate cortex, the prefrontal cortex and the nucleus accumbens^9^. In primates, *OXTR* is also expressed in the superior colliculus, the pulvinar, and the primary visual cortex, where oxytocin functions as a modulator of visual processing and allocation of attention^10^. Thus, variations in *OXTR* expression seem critical for the diversity of social behaviors across and within mammal and in particular primate species, including humans. Indeed, inter-individual variations in *OXTR* expression levels were shown to be associated with resilience to the effects of neonatal isolation on adult social attachment in female prairie voles^11^.

Several clinical trials have studied intranasal oxytocin as a tentative ASD therapeutic for improving social and behavioral function in children, adolescents, or adults diagnosed with ASD, albeit with mixed outcomes^12–22^. A follow-up study^23^ of one of these published clinical trials^12^ reported correlations of treatment efficacy with the *OXTR* single nucleotide polymorphisms (SNPs) rs53576 and rs2254298; another oxytocin trial reported efficacy correlations with *OXTR* rs6791619^13^. The large variation in these clinical trials could in-part reflect genetic or epigenetic variations in the expression levels of the oxytocin receptor gene, *OXTR*. In addition to oxytocin, the closely similar peptide hormone arginine-vasopressin is also implicated in social function via its V1AR receptor, encoded by *AVPR1A*^24–27^. Another peptide hormone studied as a tentative ASD therapeutic is insulin-like growth factor 1 (IGF-1), encoded in humans by *IGF1*. A single small study with recombinant human IGF-1 reported favorable response in ASD children^28^ and another study (NCT01970345) is ongoing.

We hypothesize that variations in *OXTR*, *AVPR1A* and *IGF1* genes could explain the variation in response to these therapeutic agents in clinical trials. To explore this hypothesis we examined *OXTR*, *AVPR1A* and *IGF1* expression levels in peripheral blood mononuclear cells (PBMC) samples from 63 ASD children, their non-ASD siblings, and matched neurotypical children aged 3–16 years (Table 1). We explore gene expression because we believe that expression levels, rather than SNPs, may afford a more robust precision medicine tool for ASD patients, as gene expression levels, unlike SNPs, also reflect epigenomic effects on transcription^29,30^. To supplement our gene expression data, we applied datamining of published Gene Expression Omnibus (GEO) datasets for exploring *OXTR* and *AVPR1A* expression variations in blood and postmortem brain samples from ASD and matched controls, respectively. Our findings suggest that PBMC expression levels of *OXTR*, *AVPR1A* and *IGF1* correlate with several standardized measures of behavior and development, including the Aberrant Behavior Checklist (ABC), Vineland Adaptive Behavior Scale (VABS), Social Responsiveness Scale (SRS), and Childhood Behavior Checklist (CBCL). Based on our findings we suggest that the PBMC expression levels of these genes should be evaluated as tentative prognostic biomarkers by clinical trials with oxytocin or other tentative ASD therapeutics.

**Table 1:** Participant demographics and behavioral scores.

Code	Age	Sex	VABS Social	SRS Total	ABC Total	CBCL Total
**Controls**						
C001	6.42	M	126	NC	NC	NC
C002	2.66	F	NC	NC	NC	NC
C003	4.71	F	NC	NC	NC	NC
C004	6.49	M	103	46	22	51
C005	6.89	F	114	82	59	75
C006	7.38	F	132	NC	NC	NC
C007	7.45	M	127	NC	NC	NC
C008	7.9	M	132	45	NC	NC
C009	12.42	M	112	46	0	42
**Healthy Siblings**						
S001	4.2	M	110	42	30	46
S002	6.62	F	NC	NC	NC	NC
S003	7.47	M	NC	NC	NC	NC
S004	7.71	F	127	44	1	54
S005	9.1	F	110	50	6	38
S006	9.1	M	112	49	9	45
S007	11.39	M	107	36	6	45
S008	12.7	F	118	45	8	52
S009	13.88	F	NC	NC	NC	NC
S010	14.77	F	112	40	1	24
S011	15.89	M	130	54	12	60
**ASD**						
A001	2.96	M	78	59	11	54
A002	3.63	M	65	86	67	73
A003	3.76	M	77	62	28	57
A004	4.42	M	74	82	21	66
A005	4.44	M	55	87	46	67
A006	4.87	M	72	89	59	74
A007	5.02	F	88	71	50	59
A008	5.18	M	55	86	64	63
A009	5.21	M	66	86	96	76
A010	5.44	M	65	66	53	59
A011	5.6	M	55	90	108	77
A012	5.62	M	61	83	77	83
A013	5.66	M	63	87	68	78
A014	5.68	M	79	69	5	51
A015	7.2	M	66	85	83	78
A016	7.34	F	73	76	61	62
A017	7.44	M	66	64	17	51
A018	8.06	F	59	90	124	75
A019	8.28	M	108	53	18	69
A020	8.38	M	53	90	97	76
A021	8.65	M	87	90	45	57
A022	8.72	M	48	90	81	64
A023	8.82	M	78	81	63	NC
A024	8.88	F	55	90	111	64
A025	8.98	M	73	88	77	73
A026	9.37	F	71	88	49	62
A027	9.55	M	47	83	58	64
A028	10.39	M	100	45	25	53
A029	10.56	M	76	85	56	75
A030	10.58	M	116	70	23	62
A031	11.11	M	50	82	38	70
A032	11.65	M	42	85	64	78
A033	11.96	M	62	71	17	54
A034	13.24	M	80	73	44	60
A035	13.65	M	72	81	24	NC
A036	13.7	M	43	86	39	61
A037	13.76	M	43	90	80	72
A038	14.16	F	74	90	81	72
A039	14.36	M	54	90	121	78
A040	14.42	F	60	90	64	NC
A041	14.49	M	57	90	35	65
A042	15.35	M	46	85	45	69
A043	16.42	F	58	90	72	74

Participant demographics along with their VABS, SRS, ABC, and CBCL scores are listed (see “Behavioral Measurements”). NC, not collected.

## Results

**Figure 1:**
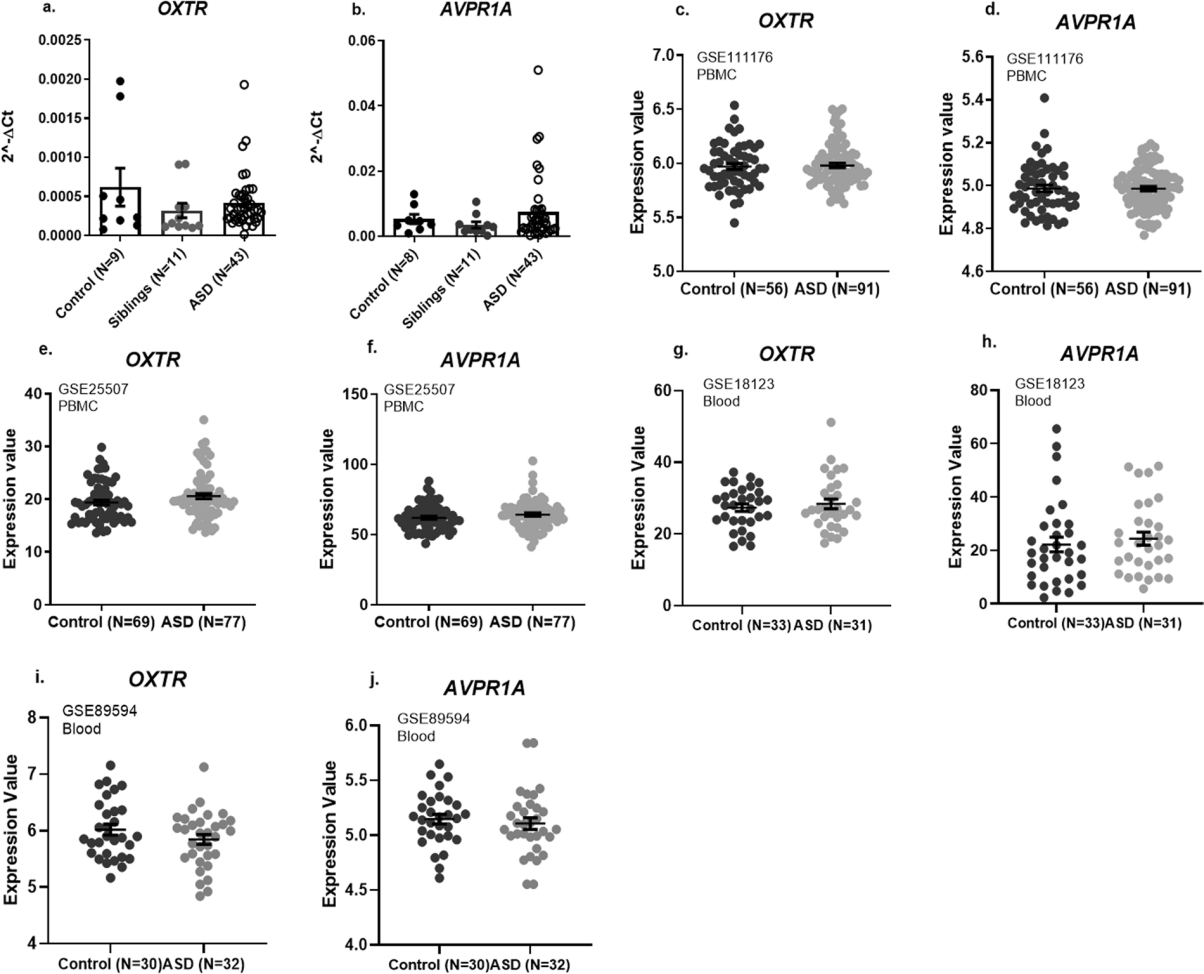
Lack of differences in PBMC mRNA expression levels of *OXTR* and *AVPR1A* compared in ASD children, their neurotypical siblings, and neurotypical age-matched children. Findings are shown for our real-time PCR findings (**a**,**b**). Corroborating our findings, no differences between ASD and control children were found in Gene Expression Omnibus (GEO) datasets from PBMC (**c**–**f**) or whole blood (**g**–**j**) samples analyzed by datamining for their *OXTR* and *AVPR1A* expression. GSE codes and cohort sizes are shown. Note the large distribution of expression levels for *OXTR* and *AVPR1A* in both our PBMC samples (**a**,**b**) and the GEO datasets (**c**–**j**). Between-group differences were analyzed by one-way ANOVA test (**a**,**b**) and by Student’s t-test (**c**–**j**).

The age, sex, and key ASD scores of study participants are presented in Table 1. As both oxytocin and vasopressin are implicated in ASD^1–3,24,25^, we searched for expression differences of the genes which code for their receptors, *OXTR* and *AVPR1A*, in PBMCs collected from the study participants. No statistically significant differences were found for the PBMC mRNA expression levels of *OXTR* or *AVPR1A* between ASD children, their neurotypical siblings, and/or neurotypical age-matched children (Fig. 1a,b). Data mining of GEO datasets derived from ASD and control PBMC (GSE111176, GSE25507) or whole blood (GSE18123, GSE89594) confirmed the lack of differences in *OXTR* or *AVPR1A* expression levels in PBMC or whole blood from ASD and neurotypical controls (Fig. 1c–j).

**Figure 2:**
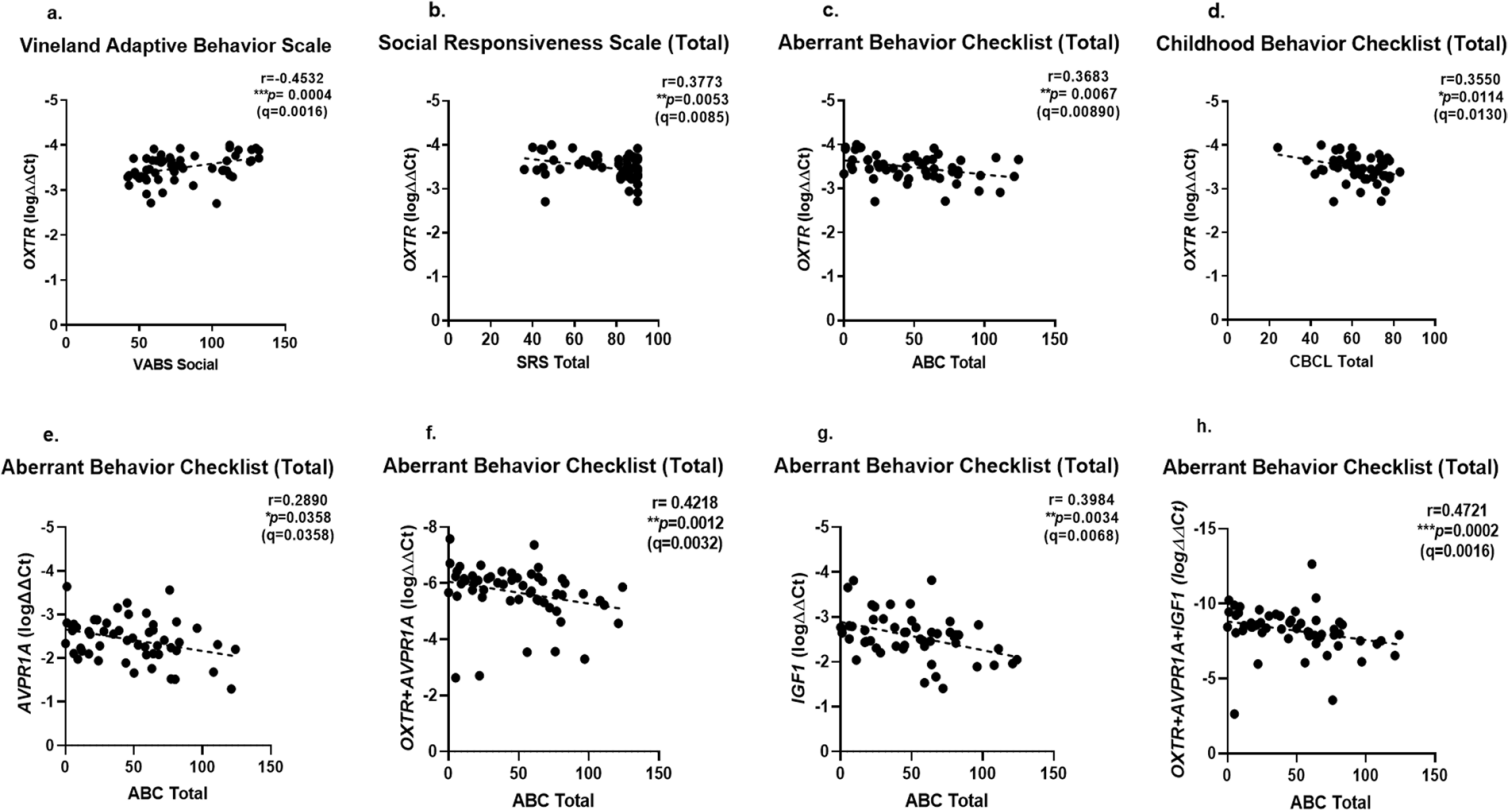
PBMC mRNA expression levels of *OXTR*, *AVPR1A* and *IGF1* correlate with ASD behavior scores. (**a**) *OXTR* vs. VABS scale (social component; N = 57); (**b**) *OXTR* vs. SRS scale (N = 53); (**c**) *OXTR* vs. ABC scale (N = 53); (**d**) *OXTR* vs. CBCL scale(N = 51); (**e**) *AVPR1A* vs. ABC scale (N = 65); (**f**) sum expression of *OXTR* + *AVPR1A* vs. ABC Scale (N = 56); (**g**) *IGF1* vs. ABC scale (N = 52); (**h**) sum expression of *OXTR* + *AVPR1A* + *IGF1* vs. ABC Scale (N = 56). The r and p values for each correlation plot (Spearman test) are shown in each panel. Dotted lines represent the linear regression lines. Note that combining PBMC mRNA expression levels of *OXTR* and *AVPR1A* yields more robust correlations compared with each gene separately; while adding PBMC *IGF1* mRNA expression levels (as a third gene) further improves the correlation with ABC scores. P values were corrected for multiple testing using Benjamini-Hochberg FDR adjustment (q-values).

Next, we searched for correlations between PBMC expression levels of *OXTR* and *AVPR1A* and the available social and behavioral scores of our study participants (see Methods; scores were recorded at the same time as blood sample collection for PBMC separation). We found that higher *OXTR* expression levels correlated with i) better development as measured by the VABS Adaptive behavioral composite, ii) less severe social impairment as measured by the SRS total t-score and iii) less severe behavior problems as measured by the lower total raw ABC scores and lower total CBCL t-scores (Fig. 2a–d). The PBMC expression of *AVPR1A* correlated with the participants total raw ABC scores, but not with their VABS, SRS, or CBCL scores (Fig. 2e; Supplementary Fig. 1e–g). Notably, no correlations with standardized scores were observed for the PBMC expression of *CD38* (Supplementary Fig. 1a–d), a gene that codes for an enzyme that synthesizes and hydrolyzes cyclic adenosine 5′-diphosphate-ribose and taking part in oxytocin secretion^31^. In addition, we also observed a correlation of the participants total raw ABC scores and their expression levels of *IGF1*, coding for insulin-like growth factor 1 (Fig. 2g**)**.

Since combining the expression levels of several genes may afford more robust diagnostic or prognostic power^32–36^ and as the ABC scores of the ASD children correlated with the *OXTR*, *AVPR1A*, and *IGF1* expression levels, we determined whether combining the expression of these genes affords a better correlation with ABC scores as compared to correlation with individual gene expression. As shown in Fig. 2h the combined expression levels of all three genes yielded improved Pearson correlations (larger r values and smaller p values) compared to each gene separately or with two-gene combinations(Fig. 2f). In order to verify the robustness of the correlation between the summed *OXTR*, *AVPR1A*, and *IGF1* expression levels with ABC scores, we randomly selected 30 individuals from our dataset. For each of 500 random selections we determined the Pearson’s correlation coefficient and p-value of the correlation between ABC score and the summed gene expression. We found that 410 (82%) of these random subsets yielded a significant correlation (p-value < 0.05) between ABC score and summed gene expression (mean Spearman’s correlation coefficient: 0.47). This is compared to 25 (5%) significant subsets expected by chance. This result demonstrates that the observed correlation is robust and is not underlined by specific outlier individuals.

## Discussion

Dysfunctional signaling by the peptide hormones oxytocin and vasopressin were suggested as contributing to ASD. While the role of these hormones has been extensively studied in several mouse models of ASD^37,38^, and oxytocin receptor knockout mice display behavioral deficits resembling autism-related behaviors^39^, such mouse models typically reflect a single mutation or deletion, and do not reflect the real-world state and wide spectrum of ASD individuals. Our study aimed to explore correlations between development and behavior in children with ASD and PBMC expression levels of the oxytocin and vasopressin receptor genes *OXTR* or *AVPR1A*, respectively.

Our findings indicate that the severity of developmental and behavioral deficits in children with ASD aged 3 to 16 years is associated with their PBMC mRNA expression levels of *OXTR* (ABC, VABS, SRS, and CBCL scores), as well as with *AVPR1A* and *IGF1* (ABC score). The PBMC expression of all three genes therefore seem to be associated with the severity of aberrant behavior in children with ASD.

Our literature search (PubMed search of May 2019) identified 11 completed and published clinical trials (listed in ClinicalTrials.gov) of intranasal oxytocin prescribed for at least 5 days and including at least 10 ASD participants who received oxytocin^12,13,15–22^ (Table 2). Only six of the 11 published studies reported a significant favorable effect of intranasal oxytocin. Notably, of the five studies with a placebo-controlled crossover design, three showed a favorable effect (Table 2). The low number of such clinical trials does not allow reaching a conclusion whether the study design (placebo-controlled crossover, placebo-controlled, or open label) affected the findings. Of note, three out of four trials with children showed favorable outcomes (Table 2) suggesting that intranasal oxytocin might be more likely to be efficacious for children. While findings from these 11 trials are difficult to interpret owing to their variable age groups, dosages, treatment durations and study designs, they clearly indicate that only some ASD individuals may benefit from oxytocin.

**Table 2:** Published clinical trials with intranasal oxytocin in ASD children and adults.

Study	N* (M/F)	Age range	Study design	Dose	Duration	Effect	*OXTR* genotyped
Kosaka 2016^13^	55 (43/12)	15–39 y	placebo-controlled crossover	16 or 32 IU/day	24 weeks*	Yes	rs6791619
Yamasue 2019^14^	53 (53/0)	18–48 y	placebo-controlled, multi-center	48 IU/day	6 weeks	No	No
Yatawara 2016^15^	31 (27/4)	3–8 y	placebo-controlled crossover	24 IU/day	14 weeks	Yes	No
Higashida 2019^16^	30 (30/0)	15–40 y	placebo-controlled crossover	16 IU/day	8 weeks	No	No
Munesue 2016^17^	29 (29/0)	15–40 y	placebo-controlled crossover	16 IU/day	8 weeks	No	No
Guastella 2015^18^	26 (26/0)	12–18 y	placebo-controlled	36 or 48 IU/day	8 weeks	No	No
Anagnostou 2012^19^	19 (16/3)	18–60 y	placebo-controlled	48 IU/day	6 weeks	Yes	No
Dadds 2014^20^	19 (19/0)	7–16 y	placebo-controlled	12 or 24 IU/day	5 days	No	No
Watanabe 2015^12,23^	18 (18/0)	24–43 y	placebo-controlled crossover	48 IU/day	6 weeks	Yes	rs53576, rs2254298
Anagnostou 2014^21^	15 (11/4)	10–17 y	open label (no placebo)	0.4 IU/kg/day	12 weeks	Yes	No
Parker 2017^22^	14 (13/1)	6–12 y	placebo-controlled	48 IU/day	4 weeks	Yes	No

The table summarizes study designs, participant demographics, and outcomes from 11 published placebo-controlled clinical trials with intranasal oxytocin for at least 5 consecutive days in ASD children and adolescents (5 trials) or adolescents and adults (6 trials). The list includes trials with PubMed-listed publications that included at least 10 ASD participants who received daily intra-nasal oxytocin and analyzed for the behavioral effects. Trials are listed by decreasing numbers (N) of participants who received oxytocin, completed the study, and were analyzed for behavioral scores prior to and following the study. For trials administering oxytocin twice per day, the total daily dose is shown. In trials showing two doses, the lower dose was assigned for participants with lower weight. *In this trial the 24 week duration includes 4 week washout period between the crossover from oxytocin to placebo or vice versa.

Of the 11 trials listed in Table 2, a single trial^22^ examined the *OXTR* expression levels, and reported lack of correlation of its expression with the effect of intranasal oxytocin on behavioral scores of children diagnosed with ASD. However, this trial measured *OXTR* expression in whole blood and compared it with the participants Social Responsiveness Scale (SRS) Total Raw Score (while we studied PBMC mRNA and report correlations with ABC scores).

**Figure 3:**
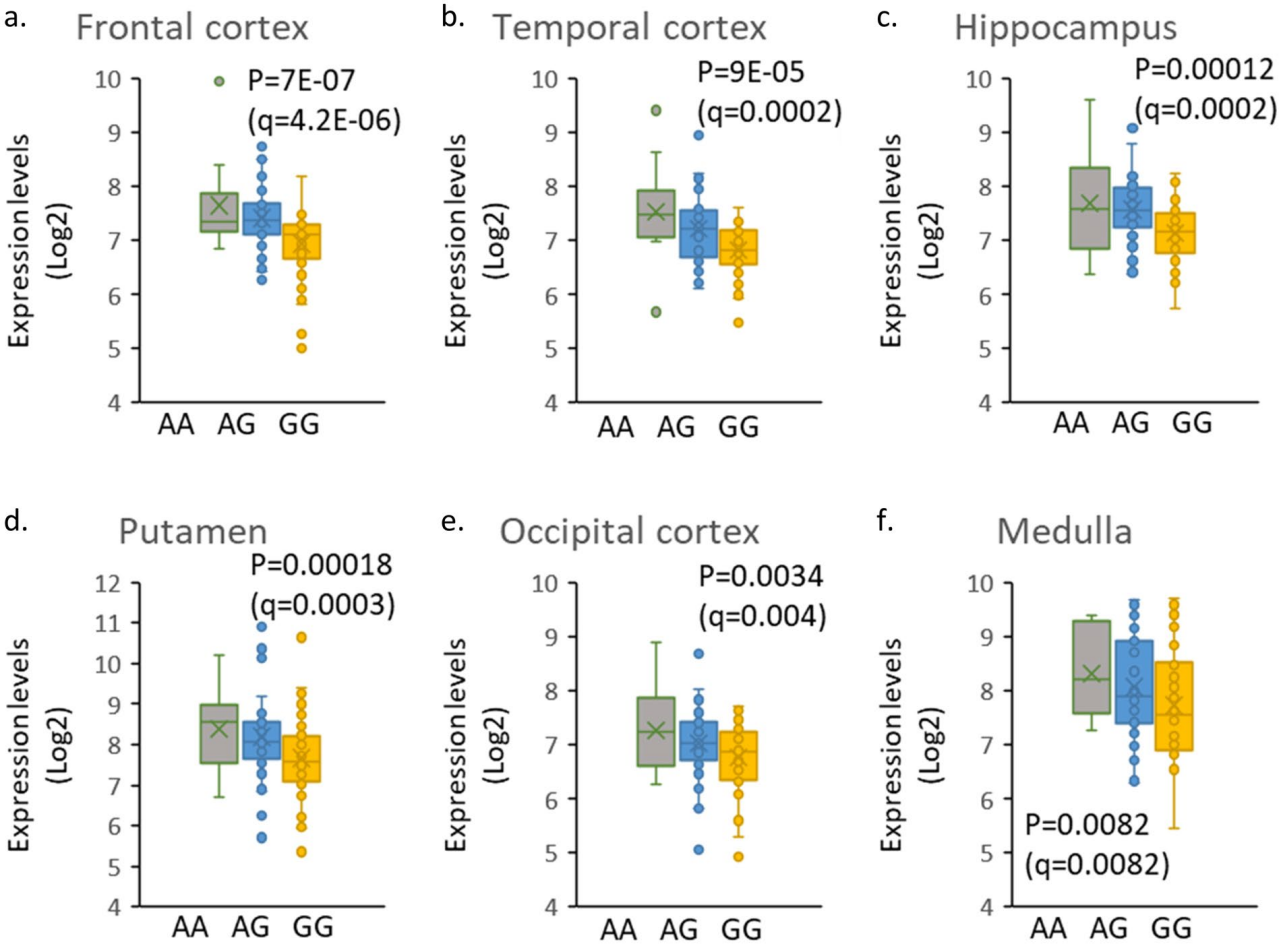
*OXTR* brain expression levels according to SNP rs53576 alleles (AA, AG, GG). Datamining was performed on the Brain eQTL Almanac website (http://www.braineac.org/). Data were derived from postmortem brain tissues of 111 neuropathologically normal individuals for the following brain regions: (**a**) frontal cortex; (**b**) temporal cortex: (**c**) hippocampus; (**d**) putamen; (**e**) occipital cortex (specifically primary visual cortex); and (**f**) medulla (specifically inferior olivary nucleus). Genotype counts were AA = 10, AG = 48, GG = 53. P-values were calculated from the eQTL data by using MatrixEQTL software (according to braineac). P values were corrected for multiple testing using Benjamini-Hochberg FDR adjustment (q-values).

Among the studies listed in Table 2 two studies examined the effects of participants *OXTR* SNPs on the efficacy of intranasal oxytocin^12,13^; both studies applied a placebo-controlled crossover design in adolescents and adults with ASD. Watanabe *et al*.^12^ reported that intranasal oxytocin has a smaller effect for participants carrying *OXTR* rs53576 or rs2254298; while Kosaka *et al*.^13^ reported lower efficacy for those carrying *OXTR* rs6791619. Of note, all three of these *OXTR* SNPs are intronic. We searched for the effects of these *OXTR* SNPs on its expression in datasets derived from studies with postmortem brain tissues (134 control individuals) deposited in the UK Brain Expression Consortium (UKBEC; http://braineac.org/) by the MRC Sudden Death Brain and Tissue Bank^40^. Our datamining showed that postmortem brain tissues from individuals carrying the minor (A) allele rs53576 allele had significantly higher *OXTR* expression levels in several brain regions, including frontal cortex, compared with tissues from GG homozygous (Fig. 3). In the general population, rs53576 was associated with general sociality^41^ and empathy^42,43^. Taken together, our current findings on poorer ABC scores in individuals with lower PBMC *OXTR* expression, along with the findings of the oxytocin clinical trial by Watanabe *et al*.^12,23^ on better efficacy (improved behavioral scores) in the *OXTR* rs53576 minor allele carriers, suggest that higher *OXTR* expression levels may correlate with improved efficacy of intranasal oxytocin in ASD individuals.

Unlike *OXTR* SNPs, *OXTR* mRNA levels seem to be more informative of ASD severity: besides DNA methylation and additional epigenetic modifiers of ASD severity^44,45^, they may inform on effects by other non-heritable effectors, such as the gut microbiome, suggested to affect ASD^46,47^. Based on our findings, we suggest that higher peripheral *OXTR* expression as detected in PBMCs may reflect differential brain expression and allow improved response to prescribed oxytocin and/or future oxytocin derivatives/agonists. The same considerations may apply also for the prognostic value of higher expression levels of *AVPR1A* and *IGF1* (Fig. 2e–h); while, being supported by the clinical trial findings by Kosaka *et al*.^13^, the predictive value seems most robust for the PBMC expression of *OXTR*. Future clinical trials with intra-nasal oxytocin, or future oxytocin derivatives such as oxytocin 5–9 or synthetic agonists^48,49^, should explore the predictive value of the participants’ PBMC mRNA of these genes. Findings from such clinical trials with ASD children or adults may allow the future stratification of ASD individuals for the specific therapeutic most likely to benefit them. Once validated by clinical trials, this may allow the future co-marketing of a drug/test combination, along the successful drug/test combinations applied for biologic therapeutics in oncology, such as the Herceptin/Her2 drug/test combination^50^. Considering the increasing rates and high societal cost of ASD, such research efforts seem timely and warranted.

## Participants, Materials and Methods

### Participants

The protocol is registered in clinicaltrials.gov as NCT02000284 and was approved by the Institutional Review Board at the University of Arkansas for Medical Sciences (Little Rock, AR). Parents of participants provided written informed consent. All methods were performed in accordance with the relevant guidelines and regulations. Children underwent a fasting blood draw in the morning. Control individuals did not have any neurological disorders or developmental delays. Thus, this study reports gene expression and social and behavioral measurements on a total of 63 children, including 43 children with ASD, 11 non-ASD siblings, and 9 unrelated neurotypical control children. This was a subset of the larger cohort of our study of mitochondrial function in children with ASD due to limited availability of PBMC for gene expression analysis. Our recent publications outline the methodology for rating participant characteristics of this participant cohort^51–53^.

### Behavioral measurements

As mentioned in our previous study^53^, our research staff was trained by a multispecialty team consisting of two licensed psychologists and a speech therapist prior to performing assessments. During the study a research psychologist supervised research staff and provided feedback and retraining if necessary. Observer rated measures included the VABS Survey Interview Form. Parents completed the ABC and the SRS. The VABS is a reliable and valid measure of the ability to perform age-appropriate everyday skills, including communication, daily living, social and motor skills, through a 20–30 minute structured interview with a caretaker^54^. Of note, functional abilities of children with autism are commonly measured with the VABS^55^ as it was in this study. Although some have used IQ to distinguish high and low functioning autism, recent studies indicate that it is a poor predictor^56^. The ABC is a 58-item questionnaire^53^ that measures disruptive behaviors and has convergent and divergent validity^57^. The SRS is a 65-item questionnaire that measures the severity of social skill deficits across five domains^58^ which has been shown to have good correspondence to the gold-standard instrument^59^. The CBCL includes demographic information and ratings of positive behaviors, academic functioning, social competence, and behavior problems commonly applied for classification of behavior disorders^60^.

### Blood collection and processing

Samples of 4 ml of venous blood were collected into an ethylenediaminetetraacetic acid-Vacutainer tube, chilled on ice and centrifuged at 1500 g for 10 minutes at 4 °C to separate plasma. Plasma was removed and stored at −80 °C for later analysis. Plasma was replaced with room temperature wash buffer containing Ca^+2^/Mg^+2^-free PBS with 0.1% BSA and 2 mM ethylenediaminetetraacetic acid. Diluted blood was then layered on top of Histopaque-1077 (Sigma Aldrich, St. Louis, MO, USA) and centrifuged at 400 g for 30 minutes at room temperature. PBMCs were collected, washed twice with wash buffer and counted using a hemocytometer.

### RNA isolation

Total RNA was extracted using RNeasy mini kit (Qiagen, Hilden, Germany) from 5 million PBMCs by following manufacturer provided protocol. RNA samples were shipped in 100% ethanol at room temperature to Tel-Aviv University. Upon arrival to Tel-Aviv, 10% 3 M Sodium Acetate and 1ul GlycoBlue™ (Thermo Fisher Scientific, MA, USA) were added and kept overnight at −80 °C. Samples were centrifuged at high speed (14,000 g, 4 °C) for 30 minutes. Pellets were dried and dissolved in 20ul DNAse/RNAse-free water.

### Real-time PCR

Real-time quantitative PCR (qPCR) reactions were performed with cDNA samples prepared from 1 μg RNA samples using qScript cDNA Synthesis Kit (Quanta Bio, MA, USA). Reverse transcription was performed using a thermal cycler over three steps (22 °C for 5 min, followed by 42 °C for 30 min and 85 °C for 5 min). Real-time PCR reactions were done with 10 μl mixtures containing 10 ng of cDNA, PerfeCTa SYBR® Green FastMix Kit (Quanta Bio, MA, USA) and Integrated DNA Technologies, Inc. (Leuven, Belgium) primers (shown below). *RPLP0* (Ribosomal Protein Lateral Stalk Subunit P0) was used as reference gene.

**Table:** 

Gene	Forward	Reverse
*OXTR*	TCGTGCAGATGTGGAGCGTCT	CATGTAGATCCAGGGGTTGC
*AVPR1A*	TTCTCGTGCCTACGTGACCT	GAGCAGGAACCCCTTTTGGA
*IGF1*	CAGTTCGTGTGTGGAGACAGGGG	GCAGCACTCATCCACGATGCCT
*RPLP0*	AGCCCAGAACACTGGTCTC	ACTCAGGATTTCAATGGTGCC

### GEO datamining

The NCBI Gene Expression Omnibus (GEO) was queried for expression data sets derived from ASD blood or PBMC samples containing cohorts of at least 30 ASD children or adults and a similarly sized age and sex matched control cohort. Data sets from human cell lines, postmortem tissues, and mouse ASD models were excluded. The identified data sets were queried for the expression levels of *OXTR* and *AVPR1A* using the GEO2R tool on the NCBI server (https://www.ncbi.nlm.nih.gov/geo/geo2r/). *OXTR* rs53576 associated brain expression levels were downloaded from the Brain eQTL Almanac website (http://www.braineac.org/). The expression levels corresponding to each *OXTR* rs53576 genotype (AA, AG, and GG) were plotted for six brain regions (Fig. 3). Of note, the above website did not yield information for the *OXTR* expression associated with rs2254298 or rs6791619 (the two other SNPs mentioned in our Discussion).

### Statistical analyses

qPCR data analysis was conducted using the GraphPad Prism v.6 (San Diego, CA, USA). Normality of data distribution was evaluated using the Shapiro-Wilk test; continuous variables between two groups were analyzed by Student’s t-test; outliers were detected by Grubbs test. Continuous variables between three groups were analyzed by one-way ANOVA test. P-values ≤ 0.05 were considered as significant. Data for correlation analysis were log transformed and Spearman correlation test was performed. P-values ≤ 0.01 were considered significant. P values were adjusted for multiple testing using Benjamini-Hochberg FDR correction^61^. R statistical software was used to test the robustness of correlations between the sum expression levels of *OXTR*, *AVPR1A*, and *IGF1* and the ABC scores (Fig. 2h). A total of 500 random subsets (each containing data from 30 individuals) were independently sampled from our data. For each random sample, the Pearson’s correlation coefficient and p-value of the correlation between ABC score and the summed gene expression was determined. The frequency of correlations with a p-value smaller than 0.05 was then calculated.

## Supplementary information

Supplementary figures
