# Supplementary material for: Peripheral Blood Mononuclear Cell Oxytocin and Vasopressin Receptor Expression Positively Correlates with Social and Behavioral Function in Children with Autism
Source: Sci Rep. 2019 Sep 17;9:13443. doi: 10.1038/s41598-019-49617-9 (PMC6748974; doi:10.1038/s41598-019-49617-9)

**Peripheral Blood Mononuclear Cell Oxytocin and Vasopressin Receptor Expression Positively Correlates with Social and Behavioral Function in Children with Autism**

Irena Voinsky^1^, Sirish Bennuri^2^, Julie Svigals^1^, *Richard E. Frye^3,4^, *Shannon Rose^2^, *David Gurwitz^1,5^

^1^Department of Human Molecular Genetics and Biochemistry, Sackler Faculty of Medicine, Tel Aviv University, Israel; ^2^ Department of Pediatrics, University of Arkansas for Medical Sciences and Arkansas Children's Research Institute, Little Rock, AR, USA; ^3^Department of Child Health, University of Arizona College of Medicine-Phoenix, Phoenix, AZ, USA; ^4^Barrow Neurological Institute at Phoenix Children's Hospital, Phoenix, AZ, USA; ^5^Sagol School of Neuroscience, Tel Aviv University, Israel.

*Authors for correspondence:

Richard E. Frye, email: rfrye@phoenixchildrens.com

Shannon Rose, email: SROSE@uams.edu

David Gurwitz, email: gurwitz@post.tau.ac.il

**Supplementary figures**

**Supplementary Figure 1**: Lack of statistically significant correlations for PBMC expression levels of *CD38* for VABS, SRS, ABC and CBCL behavioral scores (a-d) and *AVPR1A* for VABS, SRS, and CBCL behavioral scores and (e-g) in ASD children, their non-ASD children, and matched controls (aged 3-16 years). Note that PBMC expression levels of *AVPR1A* showed a correlation with ABC scores (main text Fig. 2e). P values>0.05 were considered as lack of correlation. See Methods for details on RNA extraction and real-time PCR experiments.

**Supplementary Figure 2**:  *IGF1* mRNA expression levels in children PBMCs show positive correlation with *OXTR* expression (a) and no correlation with *AVPR1A* expression (b). See Methods for further information.

**Supplementary Figure 3**: *OXTR* SNP rs53576 (chr3:8804371) brain expression levels from Brain Expression Consortium (BRAINEAC; http://www.braineac.org/). Data from 134 neuropathologically normal individuals of brain regions: frontal cortex, temporal cortex, putamen, hippocampus, occipital cortex (specifically primary visual cortex), and medulla (specifically inferior olivary nucleus). Genotype counts: AA=10, AG=48, GG=53.


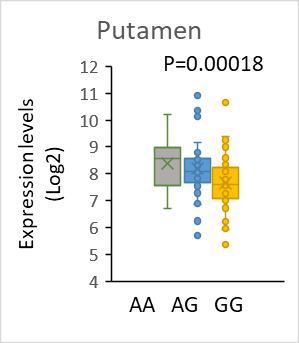

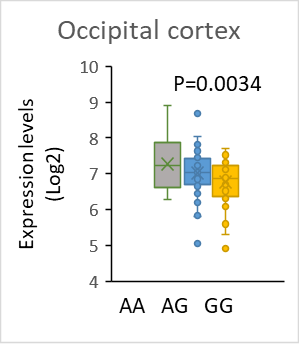

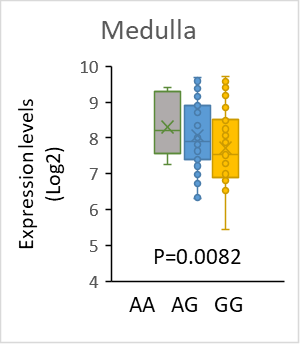

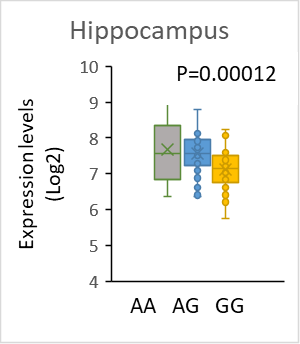

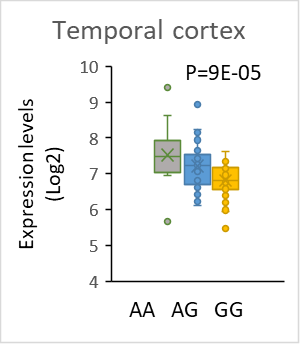

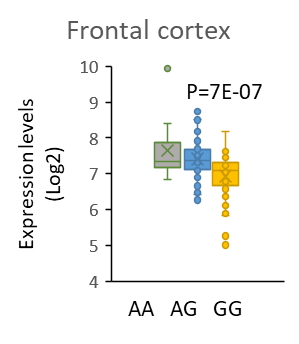

Supplement: Supplementary file 1 — Supplementary figures [file 41598_2019_49617_MOESM1_ESM.docx]
